# Clinical findings and genetic analysis of patients with copy number variants involving 17p13.3 using a single nucleotide polymorphism array: a single-center experience

**DOI:** 10.1186/s12920-022-01423-5

**Published:** 2022-12-21

**Authors:** Bin Liang, Donghong Yu, Wantong Zhao, Yan Wang, Xiaoqing Wu, Lingji Chen, Na Lin, Hailong Huang, Liangpu Xu

**Affiliations:** 1 Medical Genetic Diagnosis and Therapy Center, Fujian Key Laboratory for Prenatal Diagnosis and Birth Defect, Fujian Maternity and Child Health Hospital College of Clinical Medicine for Obstetrics & Gynecology and Pediatrics, Fujian Medical University, Fuzhou, 350001 China; 2 Fujian Obstetrics and Gynecology Hospital, Fuzhou, 350011 Fujian China; 3 Medical Research Center, Fujian Maternity and Child Health Hospital College of Clinical Medicine for Obstetrics & Gynecology and Pediatrics, Fujian Medical University, Fuzhou, 350001 China

**Keywords:** Miller–Dieker syndrome, 17p13.3 Duplication syndrome, CNV involving 17p13.3, Single nucleotide polymorphism array, Genetic analysis

## Abstract

**Background:**

17p13.3 microdeletions or microduplications (collectively known as copy number variants or CNVs) have been described in individuals with neurodevelopmental disorders. However, 17p13.3 CNVs were rarely reported in fetuses. This study aims to investigate the clinical significance of 17p13.3 CNVs with varied sizes and gene content in prenatal and postnatal samples.

**Methods:**

Eight cases with 17p13.3 CNVs out of 8806 samples that had been subjected to single nucleotide polymorphism array analysis were retrospectively analyzed, along with karyotyping, clinical features, and follow-up.

**Results:**

Eight cases with 17p13.3 CNVs consisted of five fetuses, one aborted embryo and two probands manifested severe congenital defects. The indications of prenatal testing varied considerably for the five fetuses, including ultrasound abnormalities (n = 3), segmental deletions indicated by non-invasive prenatal testing (n = 1), and intellectual disability in the mother of one fetus (n = 1). Of them, two and six harbored copy number gains and losses involving 17p13.3, respectively. The size of the detected 17p13.3 CNVs ranged from 576 kb to 5.7 Mb. Case 1 was diagnosed with 17p13.3 duplication syndrome, and cases 4, 6, and 7 with Miller–Dieker syndrome (MDS). Microdeletions of the 17p13.3 region in two cases (cases 5 and 8) involving *YWHAE* and *CRK*, sparing *PAFAH1B1*, were classified as pathogenic. Case 2 harbored a 576 kb microduplication, encompassing *YWHAE* and *CRK* but not *PAFAH1B1*, which was of maternal origin and considered a variant of uncertain significance. Case 3 carried one 74.2 Mb mosaic duplication of approximately 3.5 on chromosome 17p13.2q25.3, and two deletions at 17p13.3p13.2 and 17q25.3. The karyotype of case 3 was 46,XY,r(17)(p13q25). For five fetuses, only case 2 continued gestation and showed normal development at the age of 15 months; the others were subjected to termination of pregnancy.

**Conclusion:**

The clinical findings of 17p13.3 microdeletions or microduplications varied among subjects, and 17p13.3 CNVs often differ in size and gene content. Microdeletions or microduplications containing the typical MDS region, as well as the microdeletions involving *YWHAE* and *CRK*, could be classified as pathogenic. The clinical significance of small duplications including *YWHAE* and *CRK* but not *PAFAH1B1* remains uncertain, for which parental testing and clinical heterogeneity should be considered in genetic counseling.

**Supplementary Information:**

The online version contains supplementary material available at 10.1186/s12920-022-01423-5.

## Background

Miller–Dieker syndrome (MDS) is a rare autosomal genetic disorder caused by a ~ 1.3 Mb deletion of the 17p13.3 chromosome region, which contains *PAFAH1B1* (previously known as *LIS1*) and *YWHAE*, also known as 17p13.3 deletion syndrome, which affects approximately one in 13,000–20,000 newborns. Patients with this syndrome typically present with severe lissencephaly, dysmorphic facial features, and severe neurological abnormalities [1, 2]. Haploinsufficiency of *PAFAH1B1* (encoding LIS1) causes an isolated lissencephaly sequence. Deletions extending distally, including the gene *YWHAE* (encoding 14-3-3-epsilon), are associated with a more severe grade of lissencephaly and additional features observed in MDS [3]. Reciprocally, individuals with duplication of MDS region display variable clinical phenotypes, including structural brain abnormalities (involving the corpus callosum, cerebellar vermis, and cranial base), hypotonia, intellectual disability, a relatively distinct facial phenotype, and other variable findings [2]. Both conditions show distinct but overlapping phenotypes and often have poor prognosis, underscoring the importance of prenatal diagnosis of these disorders.

With the advent of fetal ultrasound and magnetic resonance imaging (MRI), cranial and extracranial abnormalities associated with MDS can now be prenatally identified. These abnormalities include widespread agyria, abnormal Sylvian fissure and insula, ventriculomegaly, corpus callosum dysgenesis/agenesis, microcephaly, intrauterine growth retardation (IUGR), polyhydramnios, congenital heart defects, genitourinary anomalies, micrognathia, and omphalocele [4]. However, abnormalities of the central nervous system may not always be detected by fetal ultrasound or MRI, particularly at early gestational ages. Recently, molecular genetic methods, such as chromosome microarray analysis (CMA) and BACs-on-Beads assay, have become useful for prenatally diagnosing MDS and 17p13.3 duplication syndrome [4–10]. In clinical samples, these microdeletions or microduplications involving 17p13.3 detected by CMA often differ in size and gene content. Therefore, their clinical significance requires careful interpretation. In this study, we retrospectively analyzed eight patients with copy number variants (CNVs) involving 17p13.3 by single nucleotide polymorphism (SNP) analysis over 4.5 years. We provide clinical and molecular data of patients with causal chromosomal aberrations and/or variants of uncertain significance and discuss the potential implications of phenotype-associated genes located within these CNVs.

## Materials and methods

### Patients

Eight patients with CNVs involving 17p13.3 were identified out of 8,806 samples subjected to SNP array analysis at the Fujian Provincial Maternity and Child Health Hospital from January 2016 to June 2020. These eight cases included five fetuses with various invasive diagnostic indications (cases 1–5), one aborted embryo (case 6), and two children with congenital malformations (cases 7 and 8). The clinical findings and genetic analyses of these cases included fetal ultrasound, karyotyping, SNP analysis, parental testing when possible, pregnancy outcomes, and clinical manifestations.

### SNP array analysis

Genomic DNA was obtained from the amniotic fluid, umbilical cord blood, villus, or peripheral blood and isolated using a QIAamp DNA Blood Mini kit (QIAGEN, Germany). Furthermore, CNVs were detected using the genome-wide CytoScan 750 K SNP array following the manufacturer’s instructions (Thermo Fisher Scientific Inc, Singapore). The raw data were analyzed using the Chromosome Analysis Suite (ChAS) software, version 3.1 (Thermo Fisher Scientific Inc, Singapore), and genomic imbalances were annotated based on GRCh37/hg19 Genome Build (July 2013). All CNVs were analyzed at a resolution of 100 kb/50 markers. The laboratory reported microdeletions or microduplications > 400 kb. For patients with abnormal microarray results, parental testing was performed, where possible, to determine the inheritance pattern of the deletion and/or duplication using CMA and/or standard karyotyping.

To classify the CNVs, their type (duplication or deletion), size, location, gene content, and inheritance pattern (when DNA samples of a patient′s parents were available), as well as the patient’s phenotype and clinical data were considered. We searched several genome variant databases, including the Database of Genomic Variants (http://dgv.tcag.ca/dgv/app/home), Online Mendelian Inheritance in Man (https://omim.org/), ClinVar (https://www.ncbi.nlm.nih.gov/clinvar/), ClinGen Dosage Sensitivity Curations (https://search.clinicalgenome.org/kb/gene-dosage?page=1&size=25&search =), DECIPHER (https://decipher.sanger.ac.uk/), and published literature (http://www.ncbi.nlm.nih.gov/pubmed/). CNVs were finally classified as (1) pathogenic, (2) likely pathogenic, (3) variants of uncertain significance, (4) likely benign, or (5) benign, following the guidelines of American College of Medical Genetics and Genomics [11].

### Karyotyping

Karyotyping was performed for all prenatal cases. The samples were cultured and prepared for Giemsa banding according to standard cytogenetic protocols, and the International System for Human Cytogenetic Nomenclature (2016) was used for karyotyping and description.

### Pregnancy outcome and follow-up

Pregnancy outcomes were abstracted from the delivery records of patients in our hospital. Otherwise, follow-ups were conducted with patients before and after delivery via telephone. During follow-ups, physical (length, weight, and head circumference) and mental (major movements, minor movements, cognitive ability, and intelligence) developments of the newborns were examined.

## Results

### Clinical findings of patients

The prenatal findings varied significantly among the five fetuses. Case 1 showed a slightly fast heart rate (165 beats per minute) on prenatal ultrasound at 18 weeks of gestation, and the mother presented with intellectual disability. In the first trimester, case 2 had an isolated increased nuchal translucency (NT) (3.7 mm). Subsequently, the increased NT resolved, and no other abnormalities were found later in pregnancy. Case 3 was found to have a ventricular septal defect and dysplasia of the corpus callosum at 26^+^ weeks of gestation. In case 4, non-invasive prenatal testing indicated two segmental deletions on the short arm of chromosome 17, which necessitated an invasive prenatal diagnosis of the fetus. Case 5 exhibited multiple abnormalities, including IUGR, shallow cerebral cortex, small thymus, low conus spinalis, overlapping fingers, and polyhydramnios at 28 weeks of gestation. Case 6 was a spontaneously aborted embryo, and two children (cases 7 and 8) manifested severe congenital defects. Detailed clinical information is listed in Table 1.

**Table 1:** Eight cases found with copy number variants involving 17p13.3 by SNP array

Case no	GW/age	Specimen source	Clinical findings	Karyotype	SNP array (GRCh37)	Dup/del	Size (Mb)	No. of protein-coding genes	Relevant genes or syndrome^a^	Origin	Classification of variation	Clinical outcome
1	GW 19^+^	AF	Pregnant woman manifested with intellectual disability; Slightly rapid heart rate	46,XY	17p13.3p13.2(525_3580971) × 3	Dup	3.5	63	17p13.3 duplication syndrome	NT	P	TOP
2	GW 13	AF	Increased nuchal translucency (3.7 mm)	46,XY	17p13.3(1234403_1810127) × 3	Dup	0.576	15	*YWHAE*, *CRK*	Maternal	VOUS	Normal
3	GW 26^+^	CD	Ventricular septal defect, dysplasia of the corpus callosum	46,XY, r(17)(p13q25)	17p13.3p13.2(525_5768789) × 1, 17p13.2q25.3(5768958_80004050) × 3 ~ 4, 17q25.3(80008255_81041823) × 1	Del Mosaic Dup Del	5.7 74.2 1.0	116 1007 24	MDS, Charcot-Marie-Tooth disease, type 1A (MIM:118220), Potocki–Lupski syndrome (MIM:610883), 17q11.2 duplication syndrome, 1.4-Mb (618874), and 17q12 duplication syndrome (MIM:614526)	NT	P P VOUS	TOP
4	GW 19^+^	AF	NIPT indicated a 4.2 Mb deletion in the 17p13.313.2 and a 9.58 Mb deletion in the 17p12p11.2	46,XX	17p13.3p13.2(525_4669796) × 1	Del	4.6	84	MDS	NT	P	TOP
5	GW 28	AF	IUGR, shallow cerebral cortex, small thymus, low conus spinalis, overlapping fingers and polyhydramnios	46,XX	17p13.3(525_2158383) × 1	Del	2.1	33	*YWHAE*, *CRK*	De novo	P	TOP
6	GW 9^+^	Villus	Spontaneous abortion	NT	17p13.3p13.2(525_4931704) × 1	Del	4.9	101	MDS	NT	P	NA
7	4 years old	PB	Developmental delay, congenital lissencephaly, softening of the brain	NT	17p13.3(525_2603970) × 1	Del	2.6	40	MDS	NT	P	NA
8	8 years old	PB	Intellectual disability, began walking at the age of 5	NT	17p13.3(525_1610537) × 1, 17q25.3(77008871_81041823) × 3	Del Dup	1.6 4.0	23 82	*YWHAE*, *CRK*; *P4HB*, *ACTG1*, *BAIAP2*, *TBCD*	NT	P P	NA

GW, gestation week; AF, amniotic fluid; CD, cord blood; PB, peripheral blood; NIPT, non-invasive prenatal testing; Del, deletion; Dup, duplication; NT: not tested; P: pathogenic; LP: likely pathogenic; VOUS: variants of uncertain significance; TOP, termination of pregnancy; NA, not applicable; MDS: Miller–Dieker syndrome; IUGR, intrauterine growth retardation

^a^Relevant genes or syndrome were described referring to public genome variant databases

### Karyotyping

**Fig. 1:**
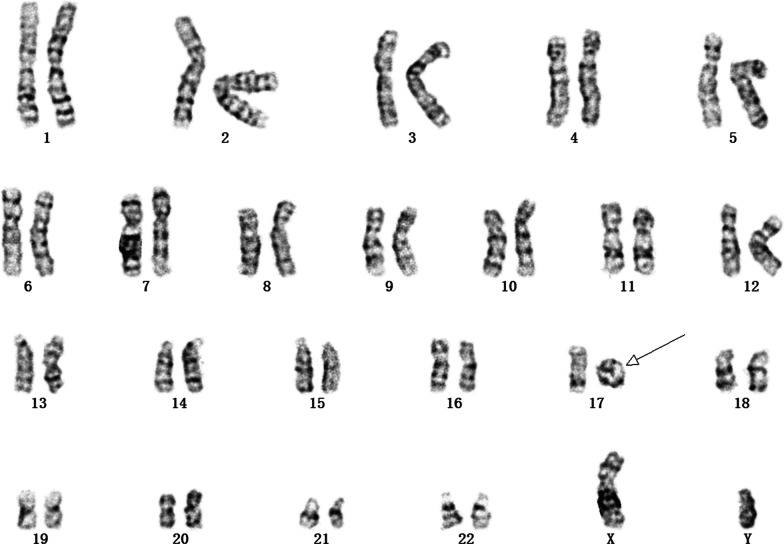
The karyotype of case 3 was 46,XY,r(17)(p13q25). The arrow points to a ring chromosome 17

Five fetuses underwent chromosomal karyotyping. Apart from case 3, no obvious abnormality was found in the other fetuses. As shown in Fig. 1, the karyotype of case 3 was 46,XY,r(17)(p13q25).

### SNP results

**Fig. 2:**
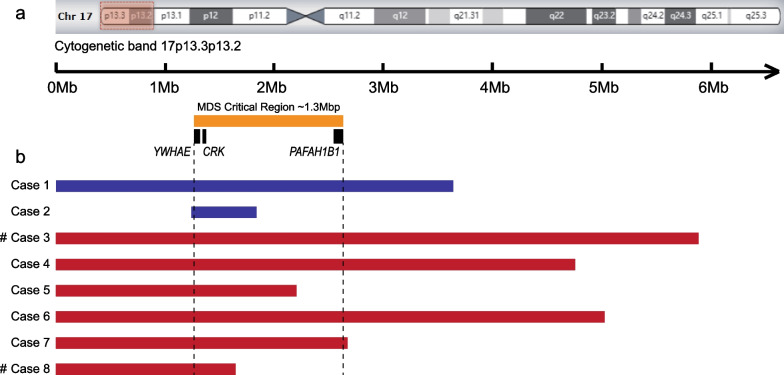
Eight cases with copy number variants (CNVs) involving 17p13.3 in our study. **a** The ideogram of chromosome 17 shows the region of interest as well as the three main genes (*YWHAE*, *CRK,* and *PAFAH1B1*). The orange bar represents the Miller–Dieker syndrome (MDS) critical region. **b** Sizes and locations of CNVs found in our patients. Red and blue bars represent copy number deletions and duplications, respectively. #: Case 3 also carried a 74.2 Mb mosaic duplication of approximately 3.5 on chromosome 17p13.2q25.3 and a 1.0 Mb deletion at 17q25.3. Case 8 also had a 4.0 Mb duplication at 17q25.3

CNVs involving 17p13.3 were detected in eight out of the 8,808 subjects, including two duplications and six deletions (Fig. 2; Additional file 1: Figure S1). The detected CNVs involving 17p13.3 ranged in size from 576 kb to 5.7 Mb; except for the microduplication in case 2, these CNVs were identified as pathogenic (Table 1). The fetus (case 2) harbored a 576 kb duplication within the 17p13.3 band, encompassing *YWHAE* and *CRK* but not *PAFAH1B1*, inherited from the mother who was healthy without apparently dysmorphism and has received an undergraduate education.

In addition, two cases (cases 3 and 8) carried other CNVs apart from 17p13.3 microdeletions. Notably, case 3 also carried a 74.2 Mb mosaic duplication of approximately 3.5 on chromosome 17p13.2q25.3 and a 1.0 Mb deletion in 17q25.3. This occurrence was indicative of the “ring chromosome 17” anomaly that was confirmed by karyotyping (Fig. 1). Case 8 also had a 4.0 Mb duplication at the 17q terminus; this duplication and deletion, occurring on the same chromosome, were most likely owing to a parental pericentric inversion.

### Pregnancy outcome

Of the five prenatal cases, only case 2 continued gestation, whereas the other fetuses were subjected to termination of pregnancy. Currently, this baby is 15 months old and has no obvious dysmorphic features. The height (80 cm) and weight (10.5 kg) are within the normal range. Motor function (major and fine movements) and mental development are normal as examined by physicians in community hospitals.

## Discussion

Genomic imbalances in 17p13.3 are mainly associated with neuronal migration disorders. A ~ 1.3 Mb deletion within the 17p13.3 region extending from *YWHAE* to *PAFAH1B1* is sufficient to cause MDS. In addition, recent studies have focused on a condition known as 17p13.3 microduplication syndrome [2, 3, 12]. Since individuals with either condition often exhibit poor prognosis, prenatal diagnosis of these genomic disorders is crucial. To investigate the clinical significance of CNVs involving 17p13.3 with varied sizes and gene content, we retrospectively analyzed the clinical data of eight cases. In addition, we discussed the potential implications of phenotype-associated genes located within these CNVs.

The CNVs involving 17p13.3 contained or overlapped with the MDS region in the eight cases. SNP array provided genetic diagnosis of MDS for cases 4, 6, and 7 and 17p13.3 duplication syndrome for case 1. Of these, case 7 displayed developmental delay (DD), congenital lissencephaly, and softening of the brain. In case 6, MDS may be the underlying cause of spontaneous abortion. In contrast, case 4 had no obvious clinical findings associated with MDS on the first-trimester ultrasound. MDS features, such as polyhydramnios, IUGR, ventriculomegaly, lissencephaly, and corpus callosum dysgenesis/agenesis, were often found in the second and third trimesters [4]. Apart from this, case 1 had no abnormalities in brain structure and no IUGR, both of which were previously described in the 17p13.3 duplication syndrome [10].

Apart from the cases with definite syndromes mentioned above, we focused on the clinical significance of CNVs involving 17p13.3 that overlapped with the MDS region. Currently, MDS is regarded as a contiguous gene deletion syndrome, and *PAFAH1B1*, *YWHAE*, and *CRK* in this region are of prime interest. *PAFAH1B1* is considered to cause an isolated lissencephaly sequence and contribute to MDS [3, 13]. *YWHAE* encodes 14-3-3ε, a phosphoserine/threonine-binding protein that plays a role in cortical development [14, 15]. In addition, *YWHAE* and *TUSC5* appear to contribute to craniofacial dysmorphism [3], while *CRK* functions in cell proliferation, differentiation, migration, and axonal growth and is a typical candidate gene for growth restriction. Moreover, *CRK* appears to be related to limb abnormalities and craniofacial dysmorphism [2, 16, 17].

Case 5 harbored a de novo 2.1 Mb deletion containing *YWHAE* and *CRK* but not *PAFAH1B1* and showed multiple abnormalities in the prenatal ultrasound. As previously reported, neurodevelopmental delay, growth retardation, craniofacial dysmorphisms, mild structural brain abnormalities, and seizures were observed in 17p13.3 deletions, including *YWHAE* and *CRK* but not *PAFAH1B1* [14, 18, 19]. Similarly, case 8 carried a 1.6 Mb deletion in the 17p13.3 region encompassing *YWHAE* and *CRK*, along with a 4.0 Mb duplication in the 17q25.3 region. Previous studies have reported that 17q25.3 duplication was related to DD, growth retardation, and multiple congenital anomalies [20, 21]. Therefore, we hypothesized that deletions and duplications may contribute to the clinical phenotype of this patient. Overall, the 17p13.3 microdeletion including *YWHAE* and *CRK* but not *PAFAH1B1* could be classified as pathogenic.

In case 2, the 17p13.3 duplication that included *YWHAE* and *CRK* was inherited from unaffected mother, and the ultrasound revealed a transient increase in NT. The fetus was then continued developing and had a good presentation at 15 months old. The duplication in case 2 can be identified as class I of 17p13.3 microduplication syndrome [3]. The individuals in this category, including three patients from Bruno et al. [3] (cases 9, 11 and 12) and four from Bi et al. [22] (subjects 1–4), had autism manifestations, behavioral symptoms, learning disabilities, subtle dysmorphic facial features, subtle hand/foot malformations, and a tendency to postnatal overgrowth, among other disorders. Another study from Curry et al. [23] described eight patients in Group 1 17p13.3 microduplications who presented with developmental, behavioral and brain abnormalities, and rare variant phenotypes such as cleft palate and split hand/foot with long bone deficiency. Regarding inheritance of these 15 patients, six were de novo, six were inherited from an unaffected parent, and three were unknown. The duplication inherited from a normal parent may be owing to reduced penetrance and variable expressivity. In addition, we searched the DECIPHER database and found 12 duplications involving *YWHAE* and *CRK*, but not *PAFAH1B1*, which was approximately 300 kb. Eight out of 12 cases lacked parental analysis and showed a wide spectrum of phenotypes not characterized by autism. Therefore, the extent of contribution of the variants to their phenotypes cannot be ascertained.

Furthermore, the likelihood of a single-gene mutation causing propositus manifestations cannot be ruled out. The two-hit model proposed by Girirajan et al. [24] suggests that a secondary disruptive event (another CNV, a point mutation or environment factors) could result in more severe clinical manifestations in neurodevelopmental diseases. Likewise, Tolezano et al. [25] investigated the genetic factors that contribute to variable expressivity of class I 17p13.3 microduplications, providing new evidence regarding the contribution of *RORA* and *DIP2B* to neurocognitive deficits such as autism and intellectual disability, respectively. Moreover, in group I 17p13.3 microduplication, Curry et al. [23] reported that disruption of *ABR* and duplication of *BHLHA9* were associated with clefts and split hand/foot with long bone deficiency phenotypes, respectively. Capra et al. [26] reported that a boy carrying a maternally inherited 329.5-kb 17p13.3 duplication, including *BHLHA9*, *YWHAE*, and *CRK*, presented with mild dysmorphic phenotype, autism, and mental retardation, while his mother was affected by a bipolar and borderline disorder and was addicted to alcohol. It can be seen that phenotypic heterogeneity existed in the mother and her child. Another report [27] described two patients manifesting distinctive features (patient 1, primary hypothyroidism; patient 2, bilateral cryptorchidism) that were not previously described in the duplication 17p13.3 spectrum. Whether these rare manifestations observed in the two patients were caused by a two-hit event or not is not known. Overall, considering 17p13.3 microduplication showing reduced penetrance, variable expressivity, and lack of a clear pathogenic mechanism, the clinical significance of the microduplication encompassing only *YWHAE* and *CRK*, but not *PAFAH1B1*, requires further investigation.

Interestingly, case 3 also carried a 74.2 Mb mosaic duplication of approximately 3.5 on chromosome 17p13.2q25.3 and a 1.0 Mb deletion in the 17q terminus, in addition to deletion of the MDS region. The SNP data were consistent with that some cells have ring 17 while others have dicentric or interlock ring 17. Given the dosage sensitivity of genes and regions involved in the three CNVs, case 3 may show compound manifestations of these known genomic disorders, such as MDS, Potocki–Lupski syndrome (MIM:610883) [12], Charcot–Marie–Tooth disease, type 1A (CMT1A, MIM:118220) [28, 29], 17q11.2 duplication syndrome, 1.4-Mb (618874) [30, 31] and 17q12 duplication syndrome (MIM:614526) [32, 33]. Notably, the karyotype of case 3 is similar to previously reported “ring chromosome 17” syndrome [34], the manifestations of which include DD, seizures, short statures, microcephaly, and muscular hypotonia, among others. In contrast, ventricular septal defect and dysplasia of the corpus callosum were observed on ultrasound at 26^+^ weeks of gestation while other features could not be detected in the prenatal ultrasound.

A total of eight cases were detected with CNVs involving 17p13.3 in our report. However, the size and number of genes involved differed considerably, particularly when mixed deletion and duplication at chromosome 17 terminations and ring chromosome 17 were observed. It is estimated that approximately 80% of MDS cases are de novo, and approximately 20% of the conditions arise from balanced chromosomal rearrangement in parents ^4^. To our knowledge, no data have been presented from large-scale case studies to calculate the frequencies of 17p13.3 microduplication, mixed deletion/duplication on chromosome 17, and ring chromosome 17 to date, indicating the need for further study.

Our study has some limitations. First, its single-center nature and small number of patients resulted in fewer detectable 17p13.3 CNVs. Second, owing to the lack of functional experiments, whether the genes inside the duplication in case 2 are overexpressed is uncertain. Third, despite its notable advantages in CNV detection, SNP array cannot detect point mutations associated with neurodevelopmental disorders. Recently, with the development of next-generation sequencing, whole-exome or whole-genome sequencing may provide clinically relevant information in cases where SNP array fails to determine the underlying cause of a neurodevelopmental disorder.

## Conclusions

The clinical findings of 17p13.3 microdeletions or microduplications varied among subjects. SNP array allowed for accurate identification of CNV syndromes. Nevertheless, identifying the clinical significance of a CNV that overlaps with the MDS region in prenatal diagnosis remains challenging. While the microdeletions that include *YWHAE* and *CRK* are likely pathogenic, the clinical significance of small duplications encompassing *YWHAE* and *CRK* but not *PAFAH1B1* remains uncertain, rendering prenatal genetic counseling difficult. Therefore, further molecular and clinical delineation of 17p13.3 microdeletions or duplications is needed to enrich published literature and databases. Combining SNP array and next-generation sequencing might provide a good option for genetic analysis in patients with the abnormalities of central nervous system.

## Supplementary Information

**Additional file 1:** Single nucleotide polymorphism array results of eight cases with 17p13.3 copy number variants identified in our study.

## Data Availability

All data generated or analyzed during this study are included in this article. The original data that support the findings of this study are available from the corresponding author upon reasonable request.

## Abbreviations

CNVs: Copy number variants
SNP: Single nucleotide polymorphism
MDS: Miller–Dieker syndrome
MRI: Magnetic resonance imaging
IUGR: Intrauterine growth retardation
CMA: Chromosome microarray analysis
NT: Nuchal translucency
DD: Developmental delay
