# Supplementary material for: Clinical findings and genetic analysis of patients with copy number variants involving 17p13.3 using a single nucleotide polymorphism array: a single-center experience
Source: BMC Med Genomics. 2022 Dec 21;15:268. doi: 10.1186/s12920-022-01423-5 (PMC9773569; doi:10.1186/s12920-022-01423-5)
Supplement: Supplementary file 1 — Additional file 1: Single nucleotide polymorphism array results of eight cases with 17p13.3 copy number variants identified in our study. [file 12920_2022_1423_MOESM1_ESM.docx]

**Case 1:**

**arr[GRCh37] 17p13.3p13.2(526_3580971)x3**


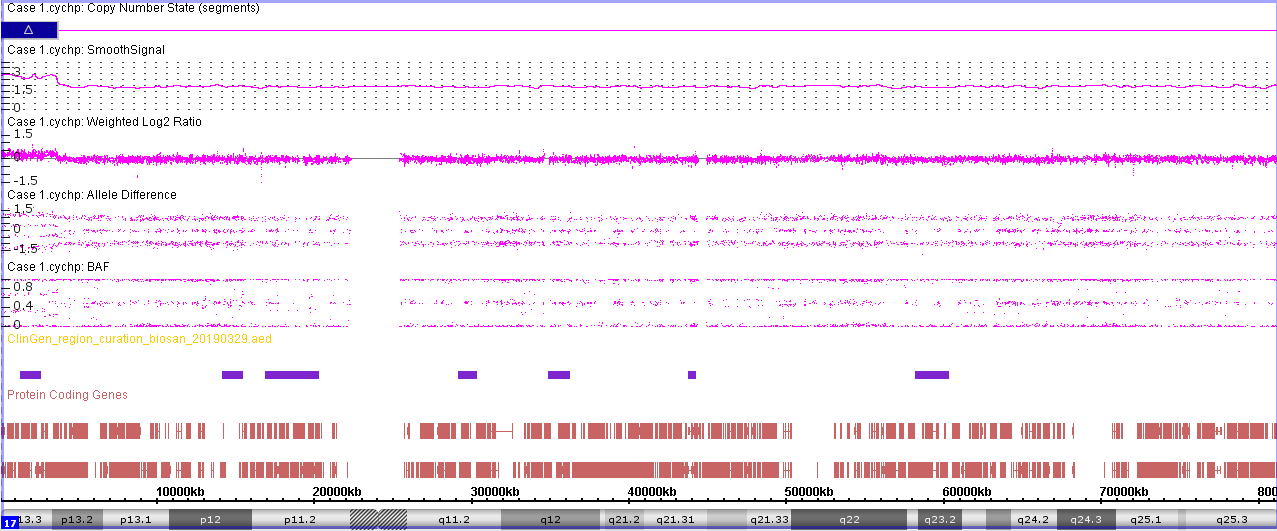


**Case 2:**

**arr[GRCh37] 17p13.3(1271914_1810127)x3**


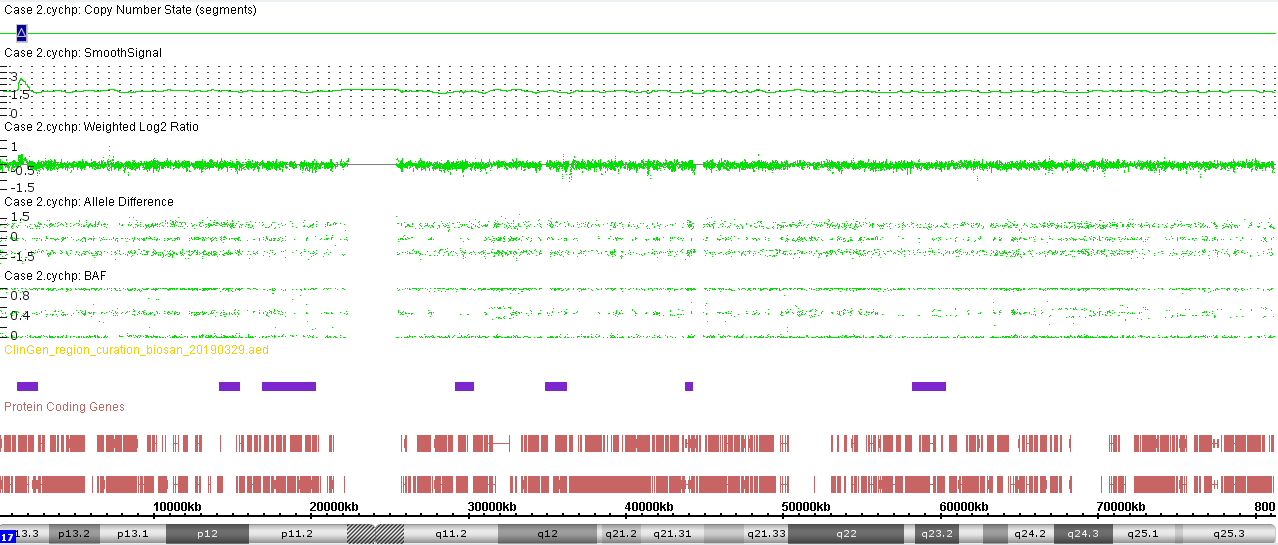


**Case 3:**

**arr[GRCh37] 17p13.3p13.2(526_5768789)x1, 17p13.2q25.3(5768959_80004050)x3~4, 17q25.3(80008256_81041823)x1**


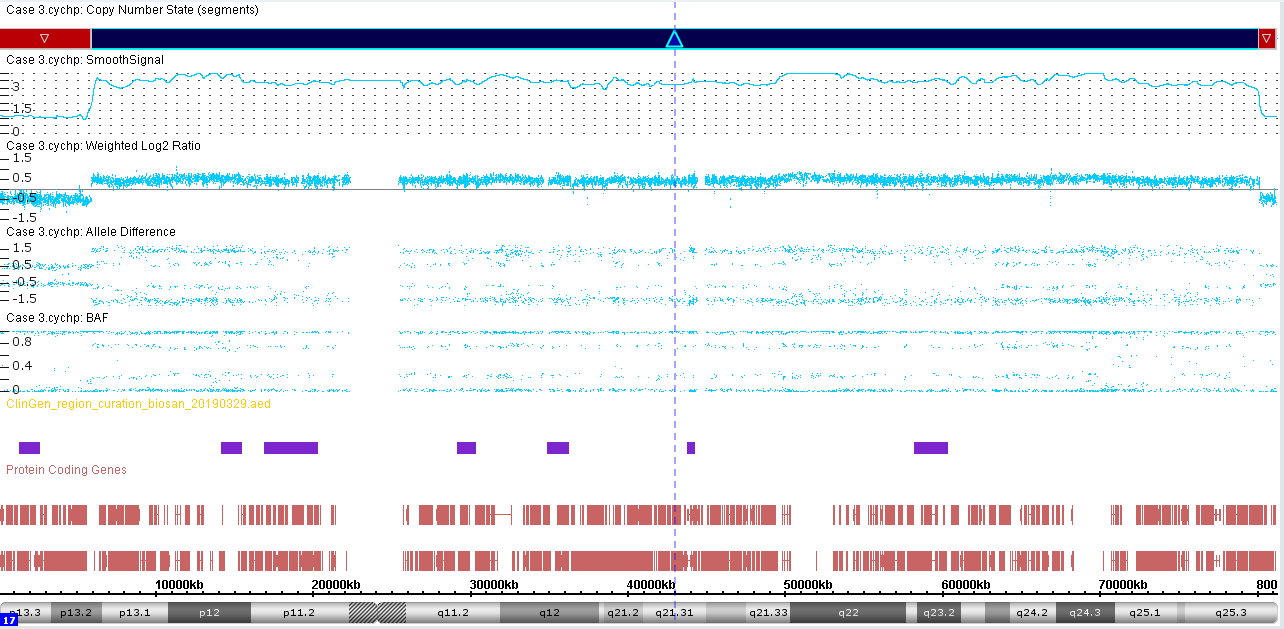


**Case 4:**

**arr[GRCh37] 17p13.3p13.2(526_4669796)x1**


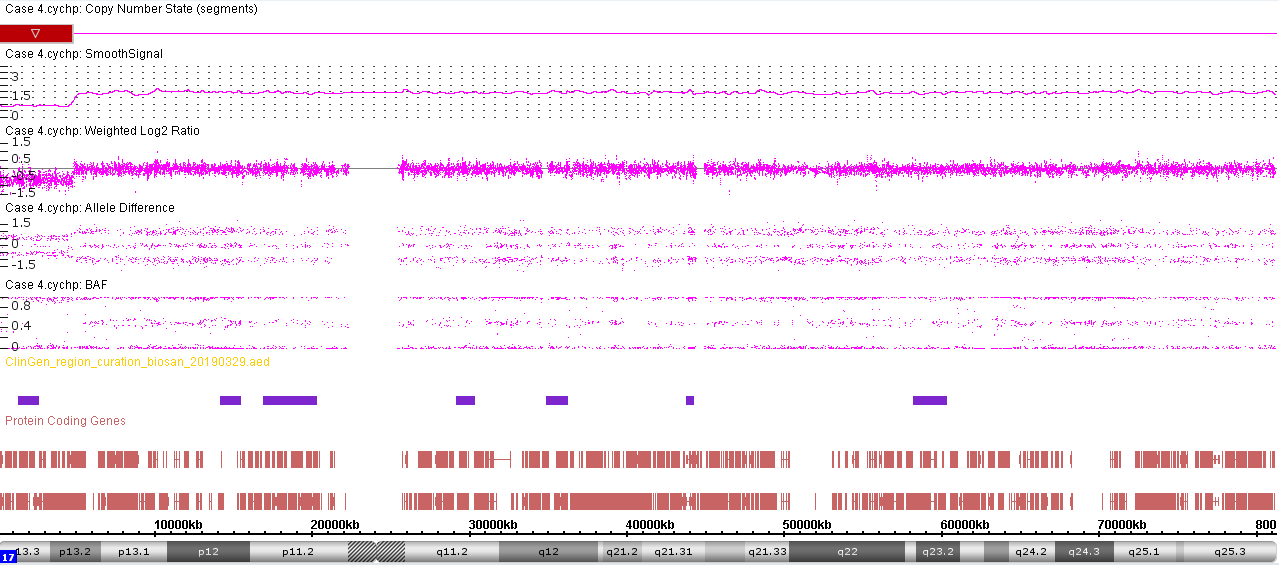


**Case 5:**

**arr[GRCh37] 17p13.3(526_2158383)x1**


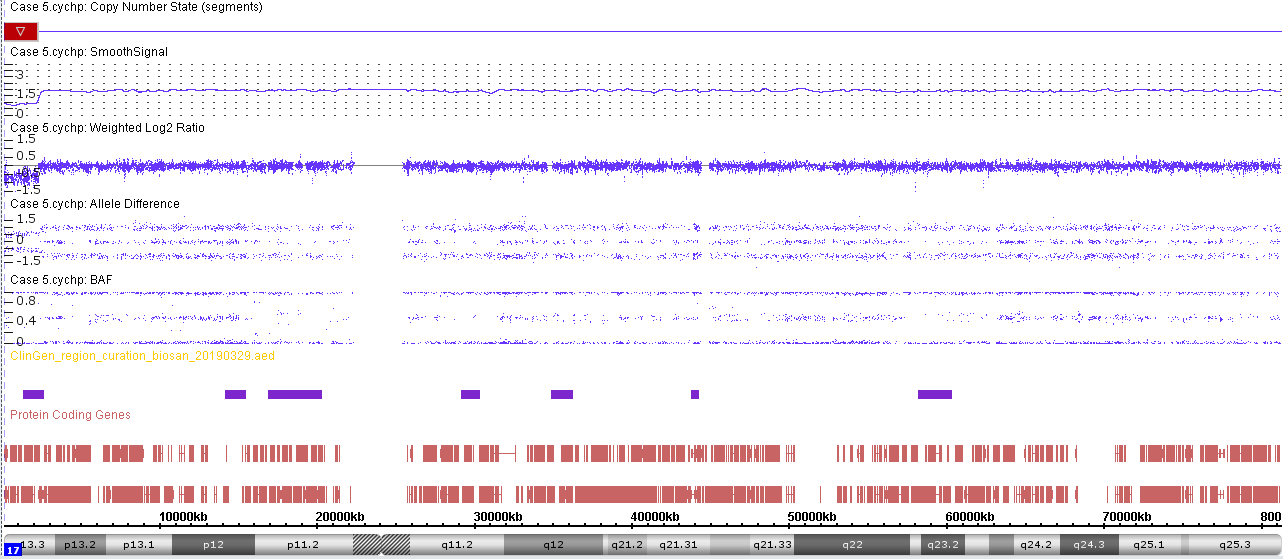


**Case 6:**

**arr[GRCh37] 17p13.3p13.2(526_4931704)x1**


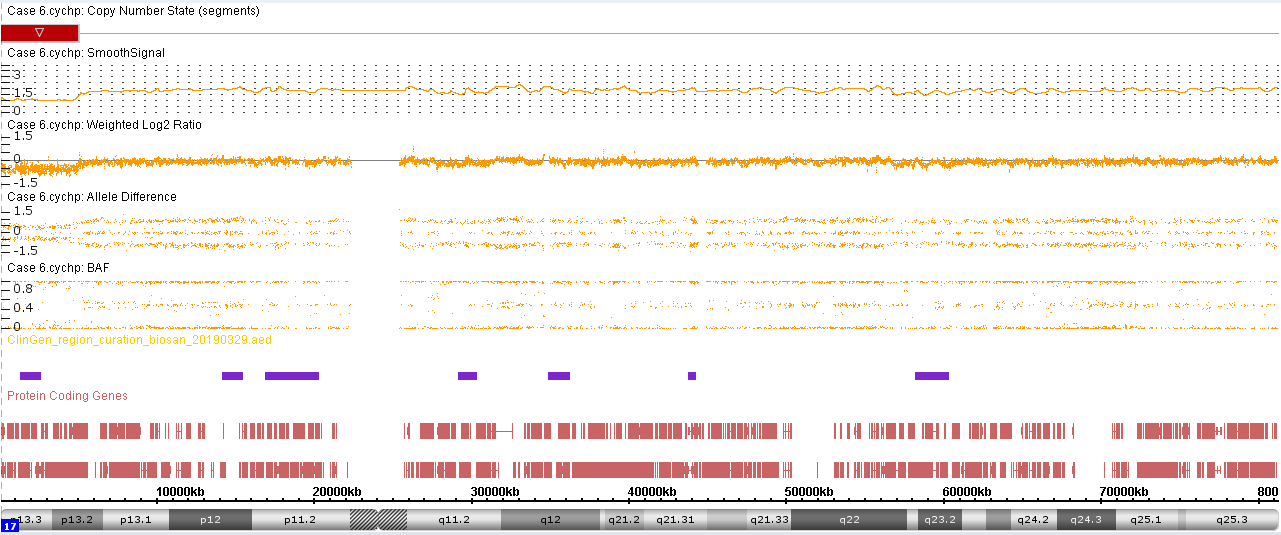


**Case 7:**

**arr[GRCh37] 17p13.3(526_2603970)x1**


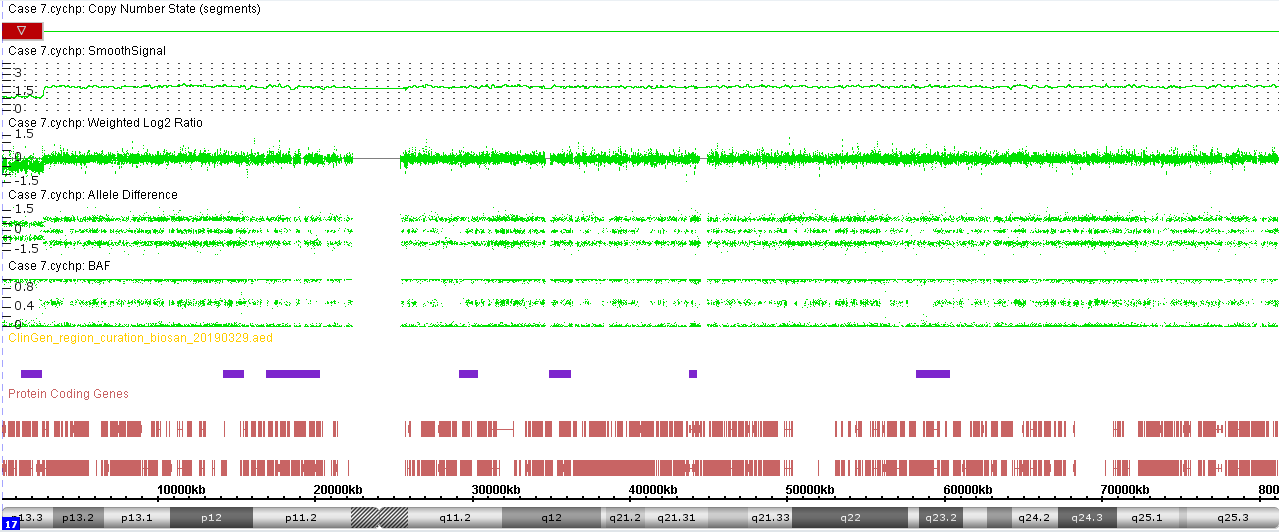


**Case 8:**

**arr[GRCh37] 17p13.3(526_1610537)x1, 17q25.3(77008872_81041823)x3**


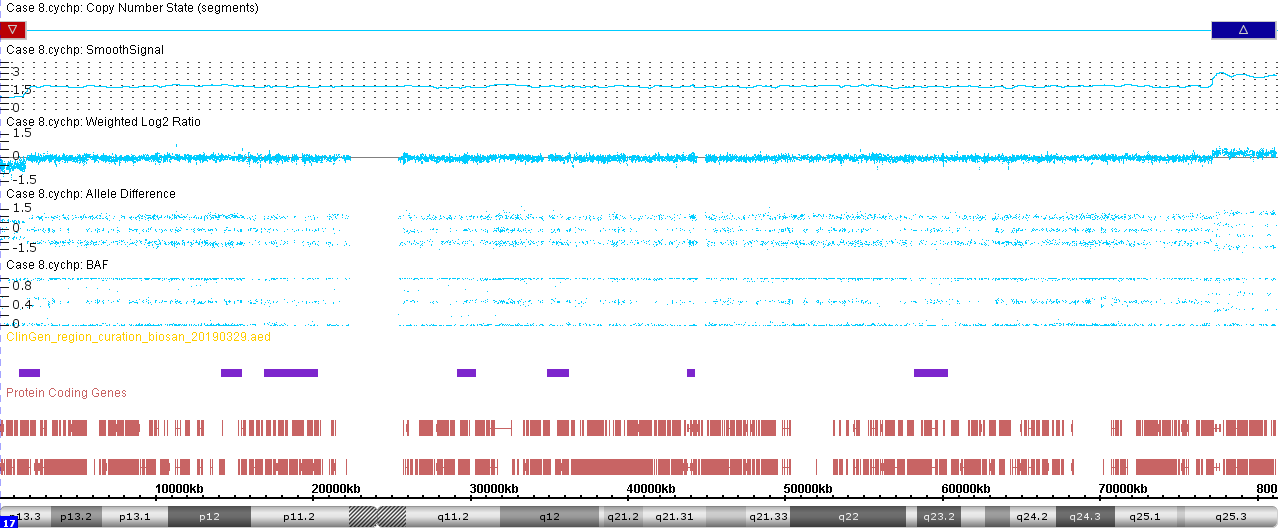


**Figure S1. Results of eight cases with 17p13.3 copy number variants (CNVs) identified in our study.** Red: loss (deletion); Blue: gain (duplication). Genomic CNVs were represented by signal lines (SmoothSignal, Weighted Log2 Ratio, Allele Difference, BAF). Some genome variant databases (ClinGen and protein Coding Genes) loaded in Chromosome Analysis Suite software (ChAS) were used for CNV classification. CNV location for each case was shown in the figure.
